# Disorder of consciousness: Structural integrity of brain networks for the clinical assessment

**DOI:** 10.1002/acn3.51729

**Published:** 2023-01-13

**Authors:** Jean Paul Medina Carrion, Mario Stanziano, Ludovico D'Incerti, Davide Sattin, Sara Palermo, Stefania Ferraro, Davide Rossi Sebastiano, Matilde Leonardi, Maria Grazia Bruzzone, Cristina Rosazza, Anna Nigri

**Affiliations:** ^1^ Diagnostic and Technology Department, Neuroradiology Unit Fondazione IRCCS Istituto Neurologico Carlo Besta Milan Italy; ^2^ Neurosciences Department “Rita Levi Montalcini” University of Turin Turin Italy; ^3^ Radiology Unit Children's Hospital A. Meyer–University of Florence Florence Italy; ^4^ IRCCS Istituti Clinici Scientifici Maugeri di Milano Milan Italy; ^5^ Department of Psychology University of Turin Turin Italy; ^6^ School of Life Science and Technology, MOE Key Laboratory for Neuroinformation University of Electronic Science and Technology of China Chengdu China; ^7^ Department of Neurophysiology and Diagnostic, Epileptology Unit Fondazione IRCCS Istituto Neurologico Carlo Besta Milan Italy; ^8^ Neurology, Public Health, Disability Unit Fondazione IRCCS Istituto Neurologico Carlo Besta; ^9^ Department of Humanistic Studies University of Urbino Carlo Bo Urbino Italy

## Abstract

**Aim:**

When studying brain networks in patients with Disorders of Consciousness (DoC), it is important to evaluate the structural integrity of networks in addition to their functional activity. Here, we investigated whether structural MRI, together with clinical variables, can be useful for diagnostic purposes and whether a quantitative analysis is feasible in a group of chronic DoC patients.

**Methods:**

We studied 109 chronic patients with DoC and emerged from DoC with structural MRI: 65 in vegetative state/unresponsive wakefulness state (VS/UWS), 34 in minimally conscious state (MCS), and 10 with severe disability. MRI data were analyzed through qualitative and quantitative approaches.

**Results:**

The qualitative MRI analysis outperformed the quantitative one, which resulted to be hardly feasible in chronic DoC patients. The results of the qualitative approach showed that the structural integrity of HighOrder networks, altogether, had better diagnostic accuracy than LowOrder networks, particularly when the model included clinical variables (AUC = 0.83). Diagnostic differences between VS/UWS and MCS were stronger in anoxic etiology than vascular and traumatic etiology. MRI data of all LowOrder and HighOrder networks correlated with the clinical score. The integrity of the left hemisphere was associated with a better clinical status.

**Conclusions:**

Structural integrity of brain networks is sensitive to clinical severity. When patients are chronic, the qualitative analysis of MRI data is indicated.

## INTRODUCTION

Neuroimaging techniques have become a critical component in the assessment of Disorders of Consciousness (DoC). DoC refers to a spectrum of conditions ranging from the coma, vegetative state/unresponsive wakefulness state (VS/UWS) to a minimally conscious state (MCS), caused by severe brain damage and presenting varying degrees of chronicity.

In the study of DoC, structural and functional MRI provide unique information on both the characterization of damaged tissue and residual brain activity. In particular, resting‐state functional MRI (rs‐fMRI) and the study of functional connectivity have provided valuable insights for understanding levels of consciousness.^1^, ^2^, ^3^ Among the brain networks, we differentiate between LowOrder networks ‐ including the sensorimotor (SM), auditory (AUD), and visual networks (lateral [LVIS] and medial [MVIS] visual) ‐ and HighOrder networks ‐ including the default mode network (DMN), salience (SAL), dorsal attention network (DAN), left and right fronto‐parietal network (L‐FP, R‐FP), and temporal network (TEMP).^4^, ^5^, ^6^, ^7^ Importantly, the 2020 European Academy of Neurology guideline recognized the contribution of rs‐fMRI in the diagnostic process, recommending that if a structural MRI exam is indicated, a rs‐fMRI sequence is suggested as part of a multimodal assessment.^8^, ^9^

Recently, also the structural integrity of brain networks has been found to be important in distinguishing between comatose patients with a good versus poor neurological outcome after 6 months^10^ and between VS/UWS versus MCS patients.^5^, ^6^ In particular, the diagnostic accuracy of DMN reached an area under the curve (AUC) = 0.72,^5^ whereas, the highest accuracy for LowOrder networks was achieved by the visual networks with AUC = 0.66, when only MRI was considered.^6^ When MRI was combined with clinical variables, namely etiology, disease duration, and age, as occurs in clinical practice, accuracy improved for all LowOrder networks (on average AUC = 0.71).^6^

When assessing structural integrity, methodological considerations become critical due to the extent and heterogeneity of lesions in DoC patients. A variety of approaches have been used, broadly classified in quantitative approaches, using automated or semi‐automated algorithms,^11^, ^12^, ^13^ and qualitative approaches, based on neuroradiologists' ratings.^5^, ^6^, ^14^, ^15^, ^16^

The quantitative methods are user‐independent, providing an unbiased measure, but are sensitive to MRI artifacts or anatomical deformations that can determine the exclusion of a potentially high number of patients (up to 35%).^11^, ^13^ Conversely, qualitative methods are time‐consuming but have the advantage of being applicable to all cases.

Another element to be considered in the characterization of DoC is etiology. In fact, the damage determined by the various etiologies^12^, ^13^ can affect the integrity of functional networks differently.^6^, ^7^, ^17^ For example, in vascular injuries, the damage may be more localized along branches of the anterior, middle, or posterior cerebral arteries and the recognition of some networks can be more challenging. In particular, it can be quite difficult to distinguish the neuronal or vascular nature of functional signals on the basis of their spatial pattern along opercular regions after hypertensive ischemic injury. By contrast, in the anoxic patients the damage is typically more diffuse and the spatial recognition of networks can be easier.^6^, ^18^

One of the crucial points to be addressed in DoC remains how structural integrity underlying the various functional networks can contribute to diagnostic classification. Indeed, the lack of integration between structural damage and abnormal brain function in DoC has recently been highlighted as one of the key gaps that need to be filled in the understanding of relevant mechanisms in these patients.^17^

This work aims to investigate the structural integrity underlying HighOrder and LowOrder brain networks in 109 chronic patients with DoC and emerged from DoC with different etiologies. The purpose is twofold: (1) to assess whether the structural integrity of the networks, both LowOrder and HighOrder, is useful for diagnostic purposes also by integrating clinical variables (i.e. age, disease duration, and etiology); (2) to assess whether a quantitative analysis can provide more information than a qualitative assessment (i.e. MRI rating) in this group of chronic patients.

## METHODS

### Participants' enrollment and assessment

A large sample of chronic adult patients with DoC and emerged from DoC (*N* = 109), admitted to a 1‐week program of multidisciplinary assessment at the Coma Research Center (CRC) of the Fondazione IRCCS Istituto Neurologico “Carlo Besta”, Milan, Italy, was enrolled and data were reported in previous studies.^5^, ^6^, ^19^, ^20^ Thus, the study included 65 patients in VS/UWS, 34 in MCS, and 10 with severe disability (SD) emerged from MCS, as assessed with the Coma Recovery Scale revised in Italian (CRS‐R)^21^, ^22^: each patient's performance was independently evaluated four times by experienced raters, with the best performance observed determining the patient's final score. Patients were also assessed with the CRS‐R‐Modified score (CRS‐R MS).^23^ Thirty‐four healthy participants (median age 39 years, range 17–66) with no history of neurological deficits were recruited as controls. The study was approved by the ethics committee of the “Carlo Besta” Institute. Written informed consent was obtained from the legally authorized representative of the patients and healthy participants.

### MRI data acquisition

MRI acquisition was performed on a 3 T MRI scanner (Achieva TX; Philips Healthcare) equipped with a 32‐channel head coil and included sagittal 3D T1‐weighted (T1w) image (repetition time [TR] = 9.86 ms, echo time [TE] = 4.59 ms, field of view [FOV] = 240 × 240 mm, voxel size = 1 mm^3^, flip angle = 8°, 185 slices) and rs‐fMRI images (gradient echo‐planar images: TR = 2.8 sec, TE = 30 ms, flip angle = 70°, voxel size = 2.5 mm^3^, matrix size = 90 × 95, 50 slices with 10% gap, ascending order, 200 volumes, eyes open). Other additional sequences included 2D T2‐weighted, 2D T2*‐weighted, FLAIR‐weighted images. Sedation was never performed. A qualitative and quantitative evaluation of the structural integrity of brain networks was performed.

### Identification of structural regions of brain networks

**Figure 1:**
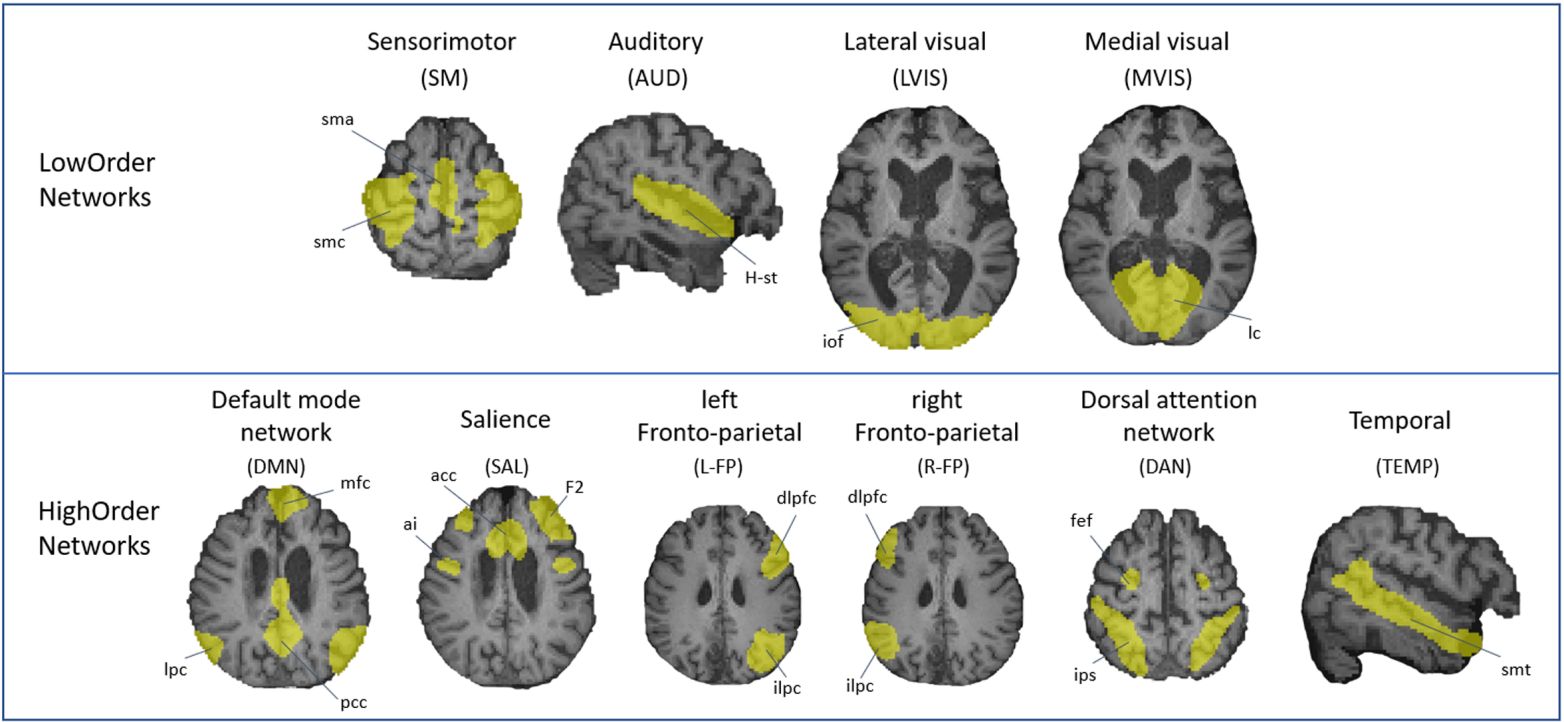
Templates of LowOrder and HighOrder brain networks. The binary mask of each brain network is overlaid on the T1w image of a MCS patient. Abbreviations: AUD, auditory network; DAN, dorsal attention network; DMN, default mode network; L‐FP, R‐FP, left and right fronto‐parietal network; LVIS, lateral visual network; MVIS, medial visual network; SAL, salience network; SM, sensorimotor network; TEMP, temporal network. The names of the nodes are reported in Table S1.

Ten brain networks were considered, divided into 4 LowOrder networks (SM, AUD, LVIS, and MVIS) and 6 HighOrder networks (DMN, SAL, L‐FP, R‐FP, DAN, TEMP). To obtain a localization of the most reliable and inclusive network areas, the network templates were derived from a group independent component analysis performed on rs‐fMRI data of healthy participants as described in our previous work^6^ using Melodic (FSL tool)^24^ (Fig. 1). The network templates were obtained from healthy participants, due to the high heterogeneity of extension and degree of damage in DoC patients.

### MRI rating

For each node of the LowOrder and HighOrder networks, anatomical corresponding areas were identified in each patient (Table S1).

Two independent expert neuroradiologists, blinded to patient information, assessed the severity of gross anatomical and signal abnormalities in anatomical regions corresponding to the selected network nodes, according to a 4‐point scale ranging from 0 to 4 (0 = extremely abnormal, i.e., parenchyma obliterated and/or pervasive hyperintensity; 1 = recognizable but distorted morphology and/or severe signal abnormality; 2 = moderate anatomical damage and/or signal abnormality; 3 = mild anatomical damage and/or signal abnormality; 4 = normal), as previously reported.^5^, ^6^, ^19^ They evaluated T1w and the additional sequences of the MRI protocol. The two neuroradiologists were expert of rs‐fMRI networks and were already familiar with the network nodes.

In cases of large disagreement, ratings were reconsidered; finally, the scores of raters were averaged for each node. Inter‐rater agreement using intraclass correlation coefficient was *P* = 0.77, indicating high reliability.^25^

### Quantitative MRI (qMRI) ‐ Gray matter analysis

**Figure 2:**
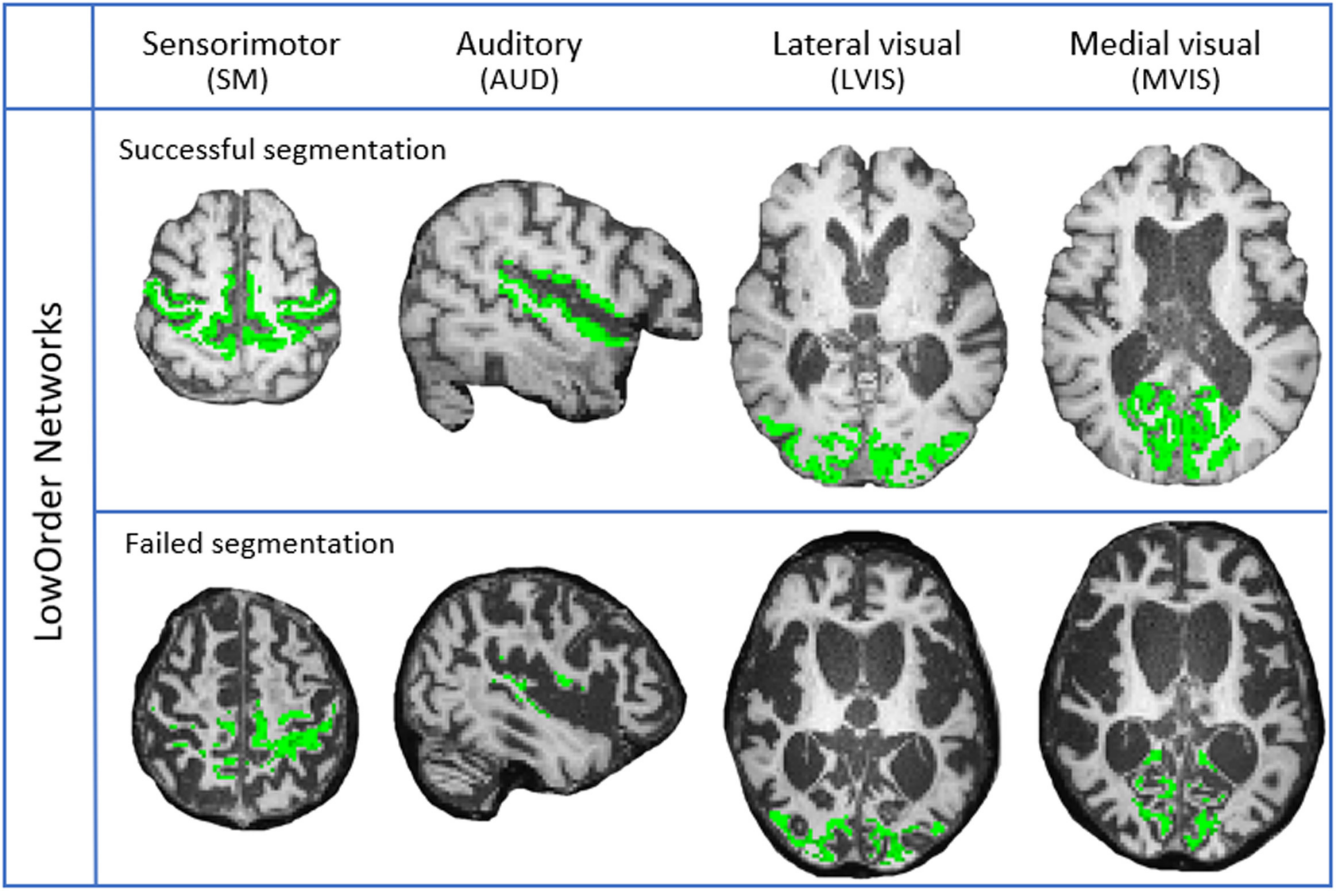
Probabilistic gray matter maps of LowOrder networks which have been thresholded in two VS/UWS patients. Abbreviations: AUD, auditory network; SM, sensorimotor network; LVIS, lateral visual network; MVIS, medial visual network. First row: patient whose segmentation was successful in all network nodes. Second row: patient whose segmentation failed in all network nodes.

To obtain a quantification of gray matter for each node of each network, T1w images were analyzed using SPM12^26^ and in‐house code running under MATLAB.^27^ For each patient, gray matter probability map, obtained from segmentation of T1w image, was subsequently normalized to the Montreal Neurological Institute (MNI) space.^26^ Due to extended brain damage, the probabilistic gray matter map was thresholded in the range 0.3–0.75 by two independent expert operators. When the probabilistic gray matter map was either overestimated or underestimated compared to the T1w image, the patient was excluded. The thresholded gray matter map was intersected with templates of HighOrder and LowOrder networks and the gray matter volume for each node of the template was extracted. Each node was visually evaluated by the two operators to determine if the estimation was accurate (Fig. 2).

### Statistical analyses

Statistical analyses were performed using R software version 4.0.3.^28^

To test VS/UWS vs. MCS differences in MRI rating for each node of each network, the Mann–Whitney U test was used and *P*‐values were false discovery rate (FDR) corrected for multiple comparisons. To test lateralization, the sum of the MRI rating of the nodes in each network per side was applied.

To test the correlation between MRI rating of each network and clinical data, (i.e., CRS‐R total score, CRS‐R MS, disease duration) and the correlation between MRI rating of LowOrder networks and the corresponding CRS‐subscale scores (SM for motor, AUD for auditory and LVIS and MVIS for visual function subscales), Spearman ρ correlation was used.

Multivariate logistic regression was performed to assess the ability to classify VS/UWS vs MCS patients. The following 4 “imaging” models were created, separately for MRI rating and gray matter analysis, using nodes as variables: (1) single network, (2) LowOrder networks, (3) HighOrder networks, and (4) the 10 networks together. Moreover, clinical variables (etiology, disease duration, and age) were considered alone in a “clinical” model and also added in each of the previous models to create “imaging + clinical” data models. The Least Absolute Shrinkage and Selection Operator (LASSO) was used to consider the best explanatory variables. The LASSO method was chosen for its ability to remove non‐relevant variables, reduce collinearity and for its less sensitivity to random errors.^29^ Leave‐one‐out cross‐validation (LOOCV) was employed to internally validate the selected model. Balanced accuracy (Bal ACCU) obtained after leave‐one‐out cross‐validation was calculated as a measure of diagnostic capability.

For each model, the sensitivity, the specificity, and the AUC, with corresponding 95% confidence interval using the bootstrap method with 2000 stratified replicates, were obtained. Following the STARD guidelines,^30^ false positive and false negative patients were also identified, and diagnostic accuracy was considered as very good for 0.8 < AUC <0.9, good for 0.7 < AUC < 0.8, sufficient for 0.6 < AUC <0.7, and bad for 0.5 < AUC <0.6. To compare regression models, the number of misclassified patients in each diagnostic category (VS/UWS and MCS) was considered.

## Results

### Participants

**Figure 3:**
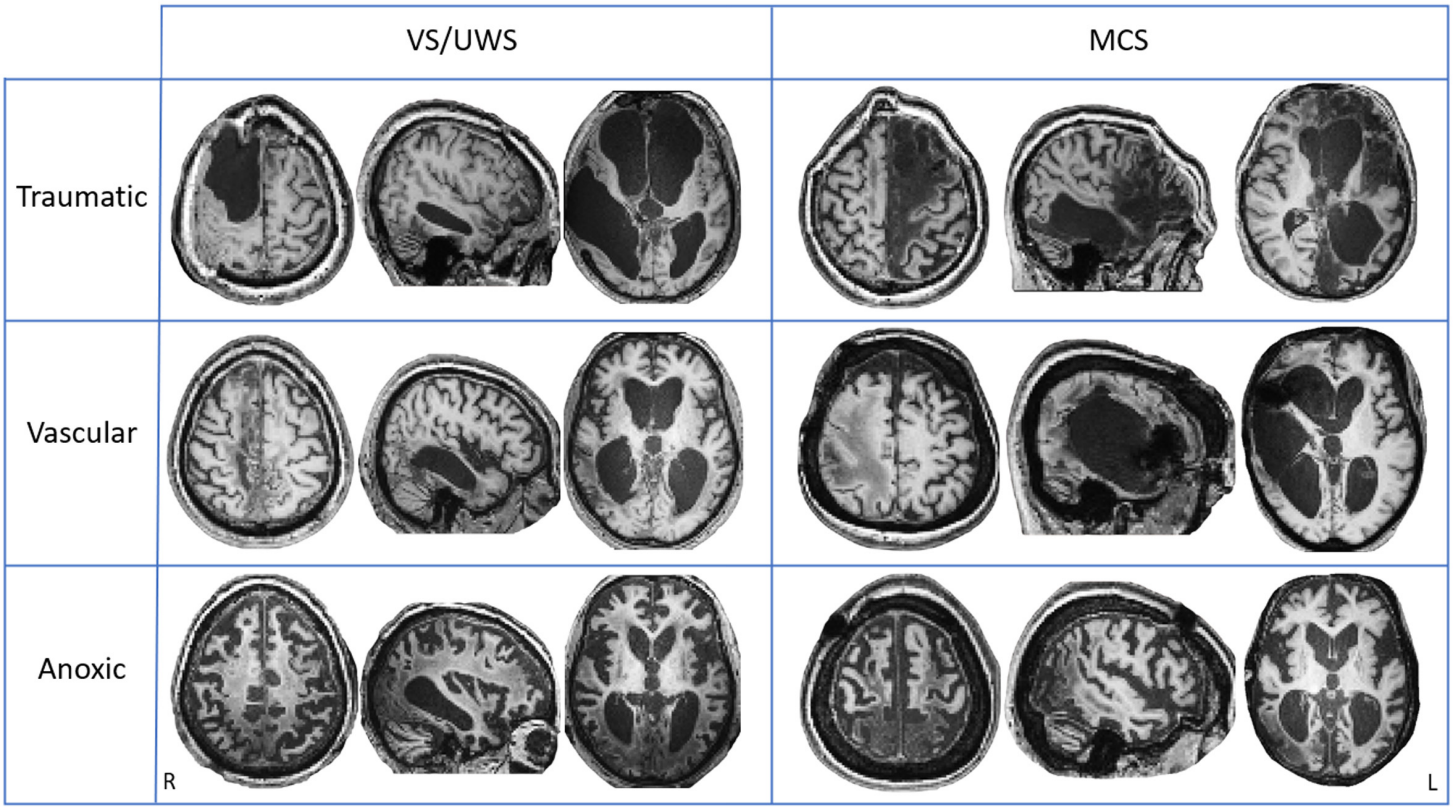
T1w images of VS/UWS and MCS patients with traumatic (48 and 82 months of disease duration, respectively), vascular (30 and 9 months of disease duration, respectively) and anoxic (16 and 209 months of disease duration, respectively) etiology. L, left; R, right.

Analyses were conducted on 33 traumatic, 39 vascular and 37 anoxic brain injury patients. The median disease duration (i.e. time post‐injury) was 27 months (range 2–252, >12 months for 82 cases), and the median age was 50 years. Table 1 presents demographic and clinical characteristics and additional data of each patient are reported in Table S2. Examples of T1w image of VS/UWS and MCS patients for traumatic, vascular and anoxic etiology were reported in Figure 3.

**Table 1:** Summary of demographic and clinical variables.

Diagnostic class	*N*	Age, yr	Sex, M/F	Etiology T/V/A	Disease Duration, mo	CRS‐R
VS/UWS	65	52 (23–79)	47/18	18/17/30	26 (3–252)	6 (3–8)
MCS	34	46 (19–83)	12/22	12/17/5	39 (6–209)	10 (7–16)
SD	10	56 (36–67)	6/4	3/5/2	14 (2–41)	18 (14–22)
All patients	109	50 (19–83)	65/44	33/39/37	27 (2–252)	7 (3–22)

Etiology is reported as traumatic/vascular/anoxic (T/V/A). Age, disease duration (i.e. time post‐injury), and CRS‐R scores are given as median (range). F, female; M, male; MCS, minimally conscious state; mo, months; *N*, number of patients; SD, severe disability; VS/USW, vegetative state/unresponsive wakefulness state; yr, years.

### Quantitative MRI (qMRI) ‐ Gray matter analysis

By grouping patients with at least one correctly segmented LowOrder network, the final sample size is 60 patients (55%), where only 21 cases (19%) had all LowOrder networks segmented, as reported in Table 2 and Figure 2. The gray matter analysis performed on LowOrder networks failed in almost half of the patients (45% of cases) due to deformation of the patient's anatomy as a consequence of severe injuries and chronicity, and poor image quality (e.g. motion artifacts), which further causes issues in segmentation. Therefore, the qMRI analysis of HighOrder networks was not performed.

**Table 2:** Results of the gray matter analysis (qMRI): the demographic and clinical variables of patients with at least one segmented network and corresponding percentages compared to the total number of patients (reported in Table 1).

Diagnostic class	*N* segmented patients / *N* of all patients (%)	Age, yr (T/V/A)	Etiology, T/V/A	Disease Duration, mo (T/V/A)	SM (T/V/A)	AUD (T/V/A)	LVIS (T/V/A)	MVIS (T/V/A)	All LowOrder (T/V/A)
VS/UWS	26/65 (40%)	51 (50/62/49)	9/10/7	24 (25/25/21)	16 (4/7/5)	20 (7/6/7)	15 (5/7/3)	16 (5/7/4)	5 (1/2/2)
MCS	24/34 (71%)	47 (41/59/47)	8/11/5	47 (55/41/44)	15 (6/6/3)	16 (4/7/5)	17 (6/8/3)	17 (5/8/4)	8 (2/4/2)
SD	10/10 (100%)	56 (57/60/51)	3/5/2	14 (19/9/14)	10 (3/5/2)	9 (3/4/1)	9 (3/4/2)	10 (3/5/2)	8 (3/4/1)
Total	60/109 (55%)	51 (47/59/49)	20/26/14	26 (24/47/14)	41 (13/18/10)	45 (14/17/14)	41 (14/20/7)	43 (13/20/10)	43 (13/20/10)

Etiology is reported as traumatic/vascular/anoxic (T/V/A). Disease duration and age are given as median values. AUD, auditory network; LVIS, lateral visual network; MCS, minimally conscious state; mo, months; MVIS, medial visual network; *N*, number of patients; SD, severe disability; SM, sensorimotor network; VS/USW, vegetative state/unresponsive wakefulness state; yr, years.

The percentage of correctly segmented patients increased with diagnosis (i.e., 40% VS/UWS, 71% MCS, 100% SD). Segmentation was successful in only 38% of anoxic patients and in 61% and 67% of traumatic and vascular patients, respectively. Reasons for segmentation failure included deformation of the patient's anatomy, chronicity of the disease (which alters the anatomy of the brain; see Fig. 3), poor image quality due to motion artifacts or valvular shunt systems, and low contrast between gray and white matter. Among 50 patients with VS/UWS and MCS, only 13 (5 VS/UWS, 8 MCS) had segmentation that was eligible for all of the 4 LowOrder networks (Table 2). No variable was considered significant to differentiate VS/UWS from MCS by the LASSO method. For the regression models with imaging and clinical variables, only clinical variables remained significant.

### MRI rating on the whole sample

**Figure 4:**
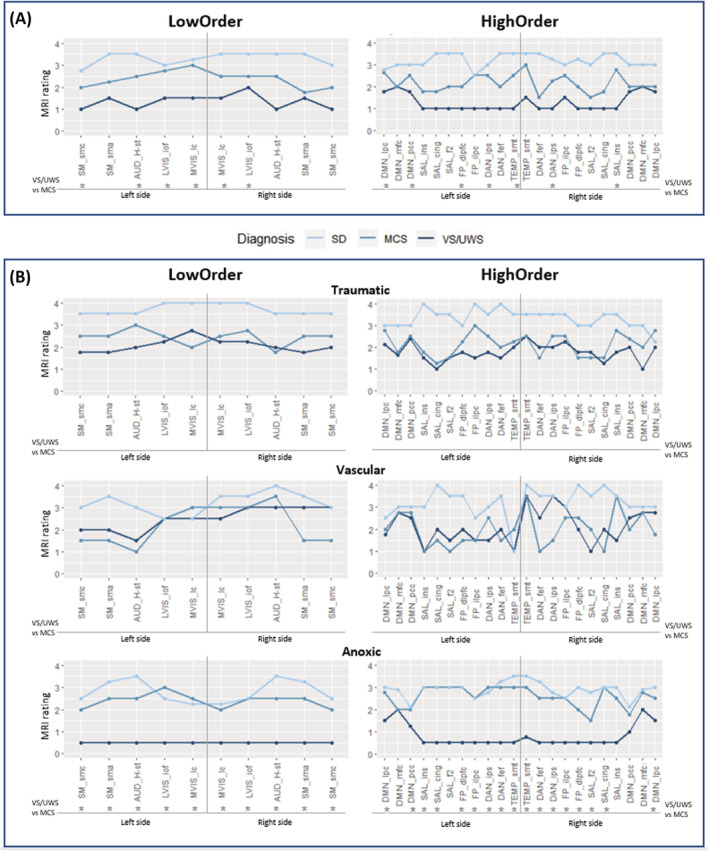
Panel A: MRI rating (median value) obtained for the different nodes of the LowOrder and HighOrder networks in the whole sample (*N* = 109). Panel B: MRI rating (median value) grouped by etiology classes. *significant differences between VS/UWS and MCS according to the Mann–Whitney U test (*P* < 0.05, false discovery rate correction applied). Abbreviations: AUD, auditory network; DAN, dorsal attention network; DMN, default mode network; L‐FP, R‐FP, left and right fronto‐parietal network; LVIS, lateral visual network; MVIS, medial visual network; SAL, salience network; SM, sensorimotor network; TEMP, temporal network. The names of the nodes are reported in Table S1.

As shown in Figure 4A, considering all networks, the level of MRI rating generally reflected the severity of diagnosis, with VS/UWS patients showing the lowest scores, followed by MCS and SD. In LowOrder networks, the difference between VS/UWS and MCS patients, observed with the Mann–Whitney U test, was significant for LVIS and MVIS networks bilaterally, and for AUD and the sensorimotor cortex of the SM network on the left side. In HighOrder networks, the difference between VS/UWS and MCS patients was significant for part of the DAN bilaterally, and part of the DMN, FP, and TEMP networks on the left side.

### Diagnostic accuracy of the MRI rating

As reported in Tables 3 and S3, in the regression models of “Imaging” data of each network, at least one imaging variable was always significant using LASSO. In particular, LowOrder networks exhibited an AUC between 0.62 and 0.66 (Bal ACCU between 0.47 and 0.56), while, including all LowOrder networks, the LASSO method considered only 3 imaging variables as significant, with an AUC of 0.70 (Bal ACCU = 0.58). Instead, HighOrder networks exhibited an AUC between 0.55 and 0.69 (Bal ACCU between 0.45 and 0.63) while including all HighOrder networks, the LASSO method considered 7 variables as significant with an AUC of 0.81 (Bal ACCU = 0.69). In the regression models with “Imaging and Clinical” variables, the imaging data were confirmed useful: in LowOrder networks, AUC ranged from 0.70 to 0.73 (Bal ACCU varied from 0.61 to 0.65), and including all LowOrder networks, the AUC was 0.74 (BAL ACCU = 0.62). In HighOrder networks, AUC ranged from 0.69 to 0.75 (Bal ACCU varied from 0.58 to 0.68), and, including all HighOrder networks, the AUC was 0.83 (BAL ACCU = 0.75). Similar results were achieved in regression models of “Imaging” and “Imaging and Clinical” variables, using both LowOrder and HighOrder networks: 10 imaging variables were considered significant with an AUC of 0.83 (Bal ACCU of 0.77 and 0.75).

**Table 3:** For the MRI rating, the diagnostic accuracy of “imaging” and “imaging + clinical variables” models (multivariate logistic regressions) for LowOrder and HighOrder networks. The analysis is performed on the sample of 99 DoC patients (65 VS/UWS + 34 MCS).

Model	MRI rating				
	Imaging		Imaging + Clinical variables		
	Bal ACCU	AUC	Bal ACCU	AUC	Clinical variables
SM	0.56	0.64	0.61	0.73	3
AUD	0.47	0.62	0.61	0.70	3
LVIS	0.52	0.66	0.65	0.71	3
MVIS	0.55	0.66	0.64	0.71	3
All LowOrder	0.58	0.70	0.62	0.74	3
DMN	0.61	0.63	0.61	0.72	3
SAL	0.63	0.67	0.68	0.75	3
L‐FP	0.60	0.64	0.61	0.73	3
R‐FP	0.45	0.55	0.60	0.69	3
DAN	0.61	0.69	0.66	0.73	3
TEMP	0.51	0.62	0.58	0.71	3
All HighOrder	0.69	0.81	0.75	0.83	3
All LowOrder + All HighOrder	0.77	0.83	0.75	0.83	3

The model with only clinical variables (etiology, disease duration and age) (i.e. “clinical” model) had balanced accuracy = 0.64 and AUC = 0.71. AUC, area under the curve; AUD, auditory network; Bal ACCU, balanced accuracy; DAN, dorsal attention network; DMN, default mode network; L, left; L‐FP, R‐FP, left and right fronto‐parietal network; LVIS, lateral visual network; MVIS, medial visual network; R, right; SAL, salience network; SM, sensorimotor network; TEMP, temporal network.

### MRI rating in etiological class

The ability to distinguish VS/UWS from MCS in terms of structural damage changed according to the etiological class (Fig. 4B): the VS/UWS vs. MCS difference was observed only in the anoxic class for both LowOrder and HighOrder networks. Descriptively, for all nodes of the LowOrder and HighOrder networks, in traumatic etiology, the MRI rating of SD patients showed higher values than VS/UWS and MCS patients, whereas, in the anoxic etiology, the MRI rating of VS/UWS patients was lower than the one of MCS and SD patients. In vascular patients, the MRI rating among diagnostic classes is generally overlapped.

### Correspondence between single networks MRI rating and CRS‐R subscales, CRS‐R total score, and disease duration on the whole sample

As reported in Table 4, considering the whole sample (VS/UWS, MCS, SD; *N* = 109), the MRI rating was significantly correlated with the CRS‐R total score for all networks, both individually and jointly (LowOrder, HighOrder, LowOrder+HighOrder); instead, the MRI rating correlated with CRS‐r MS only for AUD, LVIS, MVIS, and TEMP networks.

**Table 4:** Correspondence between single network MRI rating and CRS‐R total score, CRS‐R MS, CRS‐R subscales, and disease duration (DD) for the whole sample (VS/UWS, MCS, SD; *N* = 109).

Etiology		SM	AUD	LVIS	MVIS	All LowOrder	DMN	SAL	L‐FP	R‐FP	DAN	TEMP	All HighOrder	All LowOrder + All HighOrder
**All**	*CRS‐R total score*	0.26 **	0.31 ***	0.31 **	0.34 ***	0.34 ***	0.25 ***	0.33 ***	0.26 **	0.23 **	0.27 ***	0.32 ***	0.32 ***	0.34 ***
	*CRS‐R MS*	0.15	0.25 *	0.28 **	0.31 **	0.26 **	0.15	0.18	0.18	0.13	0.15	0.23 *	0.2	0.23 *
	*CRS‐R subscale*	0.28 *	0.26 *	0.49 ***	0.52 ***	‐	‐	‐	‐	‐	‐	‐	‐	‐
	*DD*	−0.18	−0.21*	−0.1	−0.15	−0.16	−0.24 **	−0.14	−0.16	−0.05	−0.21 *	−0.19 *	−0.19	−0.18
**Traumatic**	*CRS‐R total score*	0.23	0.19	0.28	0.27	0.27	0.29	0.26	0.15	0.18	0.14	0.17	0.02	0.25
	*CRS‐R MS*	0.15	0.11	0.16	0.09	0.16	0.29	0.11	0.08	−0.02	0.02	0.05	0.14	0.13
	*CRS‐R subscale*	0.43 *	−0.09	0.49 **	0.46 **	‐	‐	‐	‐	‐	‐	‐	‐	‐
	*DD*	−0.24	−0.23	−0.01	−0.05	−0.21	−0.19	−0.14	−0.08	−0.18	−0.29	−0.24	−0.12	−0.21
**Vascular**	*CRS‐R total score*	0.17	0.32 *	0.13	0.18	0.27	0.21	0.35 *	0.28	0.12	0.24	0.3	0.3	0.3
	*CRS‐R MS*	−0.03	0.28	0.12	0.15	0.11	−0.05	0.14	0.1	0.02	0.03	0.19	0.07	0.08
	*CRS‐R subscale*	0.17	0.47**	0.12	0.16	‐	‐	‐	‐	‐	‐	‐	‐	‐
	*DD*	−0.14	−0.15	0.16	0.17	−0.08	−0.22	−0.01	−0.15	0.21	−0.15	−0.11	−0.06	−0.07
**Anoxic**	*CRS‐R total score*	0.17	0.23	0.13	0.20	0.18	0.11	0.18	0.13	0.1	0.19	0.21	0.15	0.16
	*CRS‐R MS*	0.03	0.07	0	0.10	0.05	−0.04	0.05	0	−0.03	0.02	0.07	0	0.02
	*CRS‐R subscale*	0.1	0.36 *	0.4 *	0.52 ***	‐	‐	‐	‐	‐	‐	‐	‐	‐
	*DD*	−0.17	−0.25	−0.1	−0.15	−0.20	−0.25	−0.23	−0.22	−0.17	−0.17	−0.26	−0.24	−0.23

Correlations with CRS‐R subscales were calculated with the rating of SM for motor, AUD for auditory and LVIS and MVIS for visual function subscales. AUD, auditory network; DAN, dorsal attention network; DMN, default mode network; L‐FP, R‐FP, left and right fronto‐parietal network; LVIS, lateral visual network; MVIS, medial visual network; SAL, salience network; SM, sensorimotor network; TEMP, temporal network. Significant results are highlighted in bold; *P < 0.05; **P < 0.01; ***P < 0.001.

Moreover, the MRI rating of each LowOrder network correlated with the corresponding CRS‐R subscale, with the visual networks being the most correlated with the visual subscale (LVIS: ρ = 0.49, *P* < .001 and MVIS: ρ = 0.52, *P* < .001).

Considering the different etiologies, in traumatic and anoxic patients no significant correlation with the CRS‐R total score was observed; by contrast, in vascular patients correlation with the CRS‐R total score was significant for the AUD and SAL networks. For traumatic patients, correlation with the corresponding CRS‐R subscale scores was significant for SM, LVIS, and MVIS networks. For vascular patients, only the AUD network showed a correlation with its corresponding auditory CRS‐R subscale scores. For anoxic patients, correlation with the corresponding CRS‐R subscale scores was significant for AUD, LVIS, and MVIS networks.

Correlations with CRS‐R MS were never significant.

Disease duration did not generally have a significant effect on MRI ratings of the LowOrder and HighOrder networks, except for AUD, DMN, DAN, and TEMP.

### Lateralization

The left hemisphere of each network was always significantly different between VS/UWS and MCS patients, while the right hemisphere was significant for the LVIS, MVIS, SAL, and TEMP networks (Table 5). In both hemispheres, the VS/UWS were more damaged than MCS patients.

**Table 5:** Differences in MRI rating between VS/UWS and MCS patients calculated for the left and right hemispheres of each network with one‐tailed Mann–Whitney U test; *P < 0.05; **P < 0.01; ***P < 0.001.

Networks	Left Hemisphere			Right Hemisphere		
	VS/UWS	MCS	*U*	VS/UWS	MCS	*U*
LowOrder						
SM	2.5	4.5	699 **	2.5	4	921.5
AUD	1	2.5	783.5 **	1	2.5	891
LVIS	1.5	2.75	643***	2	2.5	759 **
MVIS	1.5	3	64.5***	1.5	2.5	696 **
HighOrder						
DMN	5	6,25	778 **	5,5	6,25	955.5
SAL	3	6	878.5 *	3	6	767.5 **
FP	2.5	4	752.5 **	3	4.5	897.5
DAN	2.5	4	750 **	2	4	925.5
TEMP	1	2.5	783.5 **	1.5	3	861 *

AUD, auditory network; DAN, dorsal attention network; DMN, default mode network; L‐FP, R‐FP, left and right fronto‐parietal network; LVIS, lateral visual network; MVIS, medial visual network; SAL, salience network; SM, sensorimotor network; TEMP, temporal network.

## Discussion

We investigated the structural integrity of 10 brain networks in 109 chronic patients with DoC and emerged from DoC with different etiologies. Dividing networks into LowOrder and HighOrder, results showed that:

- The MRI rating of the LowOrder networks did not lead to a high diagnostic accuracy between VS/UWS and MCS patients (i.e. not higher than the clinical variables), whereas the HighOrder networks, altogether, had a better diagnostic accuracy (AUC = 0.83), when clinical information was also considered. With regard to etiologies, MRI ratings showed significant differences between VS/UWS and MCS patients in the anoxic class, but not in the traumatic and vascular classes. In addition, there was a good correspondence between the MRI rating of LowOrders and CRS‐R total scores and subscales. MRI rating of the HighOrder networks also correlated with CRS‐R total score. Finally, the left side of the networks was more impaired in VS/UWS than in MCS patients.
- Regarding the two analysis approaches used, the qualitative MRI rating was more informative because it was found to be applicable indiscriminately to all patients, whereas in our sample of chronic patients the quantitative assessment was limited to a small subgroup (only 55% of cases had at least one LowOrder network segmented and only 19% had all LowOrder networks segmented).

In this work, we highlighted the importance of assessing the structural integrity of the most commonly observed brain networks to better evaluate their functionality. Undoubtedly, the lack of integration between the degree of structural damage and the associated dysfunctional activity remains one of the conceptual gaps in the study of DoC.^17^

From a methodological point of view, the qualitative approach based on the MRI rating performed by two neuroradiologists could be applied to the entire sample and all networks. In contrast, the quantitative analysis performed through gray matter segmentation could be applied to just 55% of patients, and only 19% of cases achieving a quantification of all LowOrder networks. Deformation of the patient's anatomy (e.g., extent and severity of the damage, thinning of the cortex), chronicity of the disease (which alters the anatomy of the brain compared to a standard template used for quantitative evaluation), poor image quality (due to motion artifacts or valve shunt systems, and low contrast between gray and white matter) were among the reasons for segmentation failure. As opposed to relying solely on a T1w image, the qualitative approach allowed the radiologist to integrate information from several MRI sequences. These results showed that the qualitative approach outperformed the quantitative approach, which resulted to be hardly feasible in chronic DoC patients.

At the diagnostic level, the structural integrity of networks, taken individually, had low discriminative values. Nonetheless, when structural data were combined with clinical variables, as occurs in clinical practice,^31^ the HighOrder networks achieved the highest accuracy scores, with SAL network emerging as the most accurate (AUC = 0.75), followed by DAN (AUC = 0.73), L‐FP, and DMN networks.

In normal condition, the SAL network, which encompasses the fronto‐insular region and the anterior cingulate cortex, processes interoceptive‐autonomic information and detects salient emotional stimuli, including pain.^32^, ^33^ In DoC patients, the SAL network connectivity is associated with the level of consciousness^34^, ^35^ and could distinguish between VS/UWS from MCS.^4^ In addition, the structural integrity of the SAL network resulted to be critical for the efficient regulation of DMN activity and cognitive control in traumatic brain injury patients,^36^ confirming that the interaction between HighOrder networks such as SAL, DMN, and DAN mediates the behavior.^37^, ^38^, ^39^ Further, the L‐FP network has been associated with cognitive domains such as memory, attention, language, and executive functions,^40^, ^41^ and in DoC was related to the level of consciousness.^4^, ^42^, ^43^

The MRI rating of each network correlated significantly with the CRS‐R total scores, suggesting that the level of consciousness is closely related to structural data underlying functional networks. Additional evidence for this association can be found in the significant correlation between the MRI rating of LowOrder networks and the corresponding CRS‐R subscales.

In our study, etiology was a critical factor in differentiating VS/UWS from MCS patients: on the overall sample, the significant differences between VS/UWS and MCS patients, at the level of both LowOrder and HighOrder networks, were driven by the subgroup of anoxic patients (Fig. 4). As a matter, only for this etiology it was possible to significantly distinguish VS/UWS from MCS patients. Moreover, observing the MRI ratings of the vascular etiology (Fig. 4B), patients in the 3 diagnostic categories (i.e. VS/UWS, MCS, SD) could not be easily distinguished, whereas in the traumatic etiology SD patients are always more preserved than VS/UWS and MCS patients. In vascular patients, no discrimination between VS/UWS and MCS was previously observed in the same cohort of patients on functional data.^6^ Generally, these results suggest that the differences between VS/UWS and MCS patients cannot be generalized among the different etiological classes, at least in chronic patients. Therefore, the etiology can have a crucial role in DoC, when evaluating structural,^44^ functional,^6^ and clinical^45^ data.

Furthermore, as previously reported, the left hemisphere was more damaged in VS/UWS than in MCS patients in all networks, while the right hemisphere was less sensitive to the difference between diagnoses.^5^, ^13^, ^46^, ^47^ This finding suggests that impairment to the left versus the right hemisphere has a more significant impact on the level of consciousness. This is probably because the left hemisphere is responsible for highly demanding tasks such as language and fine motor coordination.^48^ In DoC patients, language impairment can be a bias masking the presence of conscious behavior and lead to misdiagnosis.^49^, ^50^

Some limitations of our study should be noted. First, no clinical follow‐up data were collected and no prognostic analysis was performed. However, in these chronic patients, clinical progression is generally rather stable. Second, we did not consider the damage to critical subcortical regions, such as the thalamus or midbrain. However, the focus of our work was not on the key regions involved in DoC, but on the structural alteration underlying the functional networks. Finally, the post‐anoxic etiology presented an unbalanced ratio of VS/UWS versus MCS patients (30 vs. 5), which, however, is typically observed in clinical practice.

## Conclusions

This study suggests that a network‐level description of the structural damage can be a useful tool for the clinical assessment of DoC, where a qualitative MRI rating can always be employed, while a quantitative approach leads to the exclusion of too many cases when patients are chronic. The structural MRI rating of HighOrder networks, altogether, displayed a better discriminative capacity in distinguishing VS/UWS vs. MCS than LowOrder networks. When the model included imaging and clinical variables, the SAL network stood out (AUC = 0.75), followed by DAN (0.73), L‐FP (0.73), and DMN (0.72). In the future, it will be useful to integrate the structural data of brain networks to their functional activity in the characterization of DoC patients.

## Funding information

This work was supported by grant “Giovani Ricercatori” GR‐2016‐02361225 from Italian Ministry of Health (Cristina Rosazza and Anna Nigri) and RRC of Italian Ministry of Health. The Coma Research Center (CRC) project was funded by a health care grant from the Lombardy regional government (IX/000407).

## Conflicts of interest

The authors declare that the research was conducted in the absence of any commercial or financial relationships that could be construed as a potential conflict of interest.

## Author contributions

Conception and design of the study: JPM. MS, SF, CR, AN. Acquisition, analysis, or interpretation of data: JPM. MS, SF, CR, AN, LD, DS, SP, DRS, ML, MGB. Drafting of the manuscript: JPM, SP, CR, AN. Critical revision of the manuscript for important intellectual content: MS, LD, DS, SF, DRS, ML, MGB. Statistical analysis: JPM, CR, AN. Obtained funding: CR, AN, ML, MGB. Supervision: MGB, CR, AN.

## Supporting information


**Table S1.** Anatomical areas (and acronyms) underlying the 10 brain networks identified with group independent component analysis maps over healthy subjects corresponding to template.
**Table S2.** Clinical data are reported for all patients.
**Table S3.** Variables included in “Imaging” and “Imaging and Clinical” Variables for LowOrder and HighOrder networks.

## Data Availability

The data presented in this study are available on request from the corresponding author.
