# Supplementary material for: Disorder of consciousness: Structural integrity of brain networks for the clinical assessment
Source: Ann Clin Transl Neurol. 2023 Jan 13;10(3):384–96. doi: 10.1002/acn3.51729 (PMC10014003; doi:10.1002/acn3.51729)
Supplement: Supplementary file 1 — Table S1. Anatomical areas (and acronyms) underlying the 10 brain networks identified with group independent component analysis maps over healthy subjects corresponding to template. Table S2. Clinical data are reported for all patients. Table S3. Variables included in “Imaging” and “Imaging and Clinical” Variables for LowOrder and HighOrder networks. [file ACN3-10-384-s001.docx]

**Supplementary Table 1:** Anatomical areas (and acronyms) underlying the 10 brain networks identified with group independent component analysis maps over healthy subjects corresponding to template.

| **Networks** | **Nodes** | **Acronyms** |
| --- | --- | --- |
| **Sensorimotor (SM)** | *L precentral postcentral* | *smc* |
|  | *R precentral postcentral* |  |
|  | *L supplementary motor area* | *sma* |
|  | *R supplementary motor area* |  |
| **Auditory (AUD)** | *L Heschl superior temporal* | *H-st* |
|  | *R Heschl superior temporal* |  |
| **Lateral visual (LVIS)** | *L inferior occipital fusiform* | *iof* |
|  | *R inferior occipital fusiform* |  |
| **Medial visual (MVIS)** | *L lingual calcarine* | *lc* |
|  | *R lingual calcarine* |  |
| **Default  mode  network (DMN)** | *L medial frontal cortex* | *mfc* |
|  | *R medial frontal cortex* |  |
|  | *L lateral parietal cortex* | *lpc* |
|  | *R lateral parietal cortex* |  |
|  | *L posterior cingulate cortex* | *pcc* |
|  | *R posterior cingulate cortex* |  |
| **Salience (SAL)** | *L anterior insula* | *ai* |
|  | *R anterior insula* |  |
|  | *L anterior cingulate cortex* | *acc* |
|  | *R anterior cingulate cortex* |  |
|  | *L F2* | *F2* |
|  | *R F2* | *F2* |
| **L Fronto-parietal (L-FP)** | *L dorsolateral prefrontal cortex* | *dlpfc* |
|  | *L inferior lateral parietal cortex* | *ilpc* |
| **R Fronto-parietal  (R-FP)** | *R dorsolateral prefrontal cortex* | *dlpfc* |
|  | *R inferior lateral parietal cortex* | *ilpc* |
| **Dorsal  attention network (DAN)** | *L intraparietal sulcus* | *ips* |
|  | *R intraparietal sulcus* |  |
|  | *L frontal eye fields* | *fef* |
|  | *R frontal eye fields* |  |
| **Temporal (TEMP)** | *L superior medial temporal* | *smt* |
|  | *R superior medial temporal* |  |

**Supplementary Table 2**: Clinical data are reported for all patients. Diagnosis: 1=Vegetative state/unresponsive wakefulness syndrome (VS/UWS); 2=Minimally-conscious state (MCS); 3=Severe disability (SD). Etiology: 1=traumatic, 2=vascular, 3=anoxic; M = male; F = female; yr = years; mo = months.

| **Patients** | **Gender** | **Age, yr** | **Etiology** | **Disease duration, mo** | **Diagnosis** | **CRS-R total score** | **CRS-R MS** |
| --- | --- | --- | --- | --- | --- | --- | --- |
| pz_005 | M | 36 | Traumatic | 7 | SD | 18 | NA |
| pz_006 | M | 45 | Traumatic | 10 | VS/UWS | 5 | 4.5 |
| pz_007 | M | 47 | Traumatic | 19 | SD | 14 | NA |
| pz_008 | F | 44 | Traumatic | 62 | MCS | 11 | 31.93 |
| pz_009 | F | 40 | Vascular | 42 | VS/UWS | 7 | 6.59 |
| pz_011 | M | 57 | Anoxic | 16 | VS/UWS | 5 | 4.5 |
| pz_013 | F | 55 | Vascular | 23 | VS/UWS | 6 | 5.54 |
| pz_014 | M | 46 | Anoxic | 55 | VS/UWS | 8 | 6.92 |
| pz_015 | M | 65 | Traumatic | 56 | VS/UWS | 6 | 4.84 |
| pz_016 | M | 60 | Vascular | 70 | MCS | 11 | 24.64 |
| pz_017 | F | 40 | Anoxic | 44 | MCS | 11 | 24.64 |
| pz_018 | M | 43 | Anoxic | 57 | VS/UWS | 6 | 5.54 |
| pz_019 | M | 52 | Traumatic | 48 | VS/UWS | 5 | 4.84 |
| pz_020 | M | 34 | Traumatic | 40 | VS/UWS | 6 | 4.84 |
| pz_021 | M | 50 | Anoxic | 26 | VS/UWS | 6 | 4.84 |
| pz_023 | M | 77 | Traumatic | 30 | VS/UWS | 7 | 5.88 |
| pz_024 | M | 67 | Vascular | 7 | SD | 22 | NA |
| pz_025 | F | 67 | Vascular | 2 | SD | 20 | NA |
| pz_026 | M | 63 | Anoxic | 75 | MCS | 8 | 14.21 |
| pz_027 | M | 56 | Anoxic | 99 | VS/UWS | 6 | 4.84 |
| pz_028 | M | 28 | Traumatic | 82 | MCS | 16 | NA |
| pz_029 | F | 54 | Traumatic | 31 | SD | 18 | NA |
| pz_030 | F | 49 | Anoxic | 41 | VS/UWS | 8 | 6.92 |
| pz_031 | M | 49 | Anoxic | 41 | VS/UWS | 6 | 5.54 |
| pz_032 | M | 53 | Anoxic | 7 | VS/UWS | 5 | 4.5 |
| pz_033 | F | 39 | Vascular | 41 | MCS | 10 | 30.89 |
| pz_034 | M | 56 | Anoxic | 98 | VS/UWS | 7 | 5.88 |
| pz_035 | M | 59 | Vascular | 76 | MCS | 11 | 39.22 |
| pz_036 | F | 63 | Vascular | 37 | MCS | 9 | 15.26 |
| pz_038 | M | 55 | Anoxic | 33 | VS/UWS | 7 | 5.88 |
| pz_039 | M | 22 | Traumatic | 10 | MCS | 7 | 21.88 |
| pz_041 | M | 56 | Traumatic | 13 | VS/UWS | 6 | 5.54 |
| pz_042 | F | 56 | Traumatic | 25 | VS/UWS | 6 | 4.84 |
| pz_043 | F | 46 | Traumatic | 41 | VS/UWS | 7 | 5.88 |
| pz_044 | M | 66 | Anoxic | 15 | SD | 18 | NA |
| pz_045 | M | 46 | Traumatic | 27 | VS/UWS | 5 | 3.79 |
| pz_046 | M | 61 | Traumatic | 26 | VS/UWS | 3 | 3.13 |
| pz_048 | F | 57 | Vascular | 41 | SD | 22 | NA |
| pz_049 | M | 54 | Traumatic | 46 | MCS | 10 | 30.89 |
| pz_050 | F | 47 | Vascular | 59 | MCS | 14 | 56.94 |
| pz_051 | F | 43 | Vascular | 33 | MCS | 12 | 46.52 |
| pz_052 | F | 68 | Vascular | 26 | VS/UWS | 8 | 14.21 |
| pz_053 | F | 79 | Vascular | 8 | VS/UWS | 6 | 4.84 |
| pz_054 | F | 62 | Vascular | 29 | MCS | 9 | 21.51 |
| pz_055 | F | 40 | Vascular | 31 | MCS | 7 | 20.47 |
| pz_056 | M | 33 | Traumatic | 198 | MCS | 7 | 13.17 |
| pz_058 | M | 46 | Vascular | 51 | VS/UWS | 6 | 5.54 |
| pz_059 | F | 41 | Vascular | 20 | MCS | 10 | 23.59 |
| pz_060 | F | 39 | Anoxic | 141 | VS/UWS | 6 | 4.84 |
| pz_061 | F | 56 | Vascular | 22 | VS/UWS | 7 | 5.88 |
| pz_062 | M | 65 | Anoxic | 16 | VS/UWS | 7 | 5.88 |
| pz_063 | M | 52 | Anoxic | 21 | VS/UWS | 6 | 4.84 |
| pz_064 | F | 50 | Vascular | 65 | VS/UWS | 5 | 4.5 |
| pz_066 | M | 41 | Anoxic | 42 | VS/UWS | 7 | 5.88 |
| pz_067 | F | 62 | Vascular | 21 | VS/UWS | 5 | 4.5 |
| pz_068 | M | 73 | Vascular | 9 | VS/UWS | 8 | 6.92 |
| pz_069 | M | 44 | Anoxic | 12 | VS/UWS | 5 | 4.5 |
| pz_073 | M | 49 | Anoxic | 33 | VS/UWS | 7 | 5.88 |
| pz_074 | M | 60 | Anoxic | 34 | VS/UWS | 7 | 5.88 |
| pz_075 | M | 29 | Anoxic | 91 | VS/UWS | 7 | 5.17 |
| pz_076 | M | 39 | Vascular | 36 | VS/UWS | 6 | 4.84 |
| pz_077 | M | 66 | Anoxic | 26 | VS/UWS | 6 | 4.84 |
| pz_078 | M | 45 | Vascular | 30 | VS/UWS | 8 | 6.92 |
| pz_079 | M | 50 | Vascular | 19 | SD | 21 | NA |
| pz_080 | M | 49 | Traumatic | 16 | VS/UWS | 7 | 5.88 |
| pz_082 | F | 47 | Anoxic | 209 | MCS | 11 | 31.93 |
| pz_083 | M | 44 | Vascular | 48 | VS/UWS | 6 | 4.84 |
| pz_084 | F | 83 | Vascular | 119 | MCS | 8 | 13.17 |
| pz_085 | M | 52 | Anoxic | 9 | VS/UWS | 6 | 4.84 |
| pz_086 | M | 33 | Anoxic | 16 | VS/UWS | 6 | 4.84 |
| pz_088 | M | 60 | Traumatic | 43 | VS/UWS | 6 | 5.54 |
| pz_089 | M | 25 | Traumatic | 12 | VS/UWS | 6 | 5.54 |
| pz_090 | M | 44 | Anoxic | 70 | VS/UWS | 7 | 5.88 |
| pz_091 | F | 25 | Traumatic | 12 | MCS | 7 | 13.88 |
| pz_094 | M | 38 | Traumatic | 41 | MCS | 12 | 24.64 |
| pz_095 | M | 76 | Vascular | 15 | VS/UWS | 8 | 22.22 |
| pz_097 | F | 71 | Vascular | 51 | MCS | 10 | 23.59 |
| pz_098 | F | 53 | Vascular | 9 | VS/UWS | 5 | 4.5 |
| pz_100 | M | 59 | Vascular | 24 | MCS | 12 | 32.26 |
| pz_102 | M | 30 | Traumatic | 17 | VS/UWS | 6 | 5.54 |
| pz_104 | F | 58 | Traumatic | 29 | MCS | 9 | 22.55 |
| pz_105 | M | 37 | Anoxic | 12 | SD | 17 | NA |
| pz_106 | F | 37 | Vascular | 32 | MCS | 10 | 23.59 |
| pz_107 | M | 35 | Traumatic | 9 | VS/UWS | 7 | 5.88 |
| pz_109 | M | 23 | Traumatic | 14 | VS/UWS | 7 | 5.88 |
| pz_110 | M | 63 | Anoxic | 6 | VS/UWS | 6 | 5.54 |
| pz_112 | F | 61 | Anoxic | 3 | VS/UWS | 6 | 5.54 |
| pz_113 | M | 45 | Anoxic | 14 | VS/UWS | 7 | 5.88 |
| pz_114 | M | 52 | Anoxic | 146 | VS/UWS | 8 | 14.21 |
| pz_116 | F | 34 | Vascular | 9 | MCS | 11 | 31.93 |
| pz_117 | F | 39 | Anoxic | 12 | MCS | 8 | 14.21 |
| pz_118 | F | 67 | Vascular | 58 | VS/UWS | 7 | 5.88 |
| pz_120 | F | 68 | Anoxic | 7 | MCS | 10 | 23.59 |
| pz_121 | F | 82 | Vascular | 11 | MCS | 9 | 22.55 |
| pz_122 | M | 33 | Traumatic | 5 | VS/UWS | 6 | 5.54 |
| pz_123 | F | 42 | Anoxic | 25 | VS/UWS | 8 | 6.92 |
| pz_124 | M | 52 | Anoxic | 17 | VS/UWS | 7 | 5.88 |
| pz_126 | F | 69 | Vascular | 15 | MCS | 10 | 23.59 |
| pz_128 | M | 38 | Anoxic | 8 | VS/UWS | 6 | 5.54 |
| pz_129 | F | 57 | Vascular | 12 | VS/UWS | 7 | 5.88 |
| pz_130 | F | 60 | Vascular | 9 | SD | 14 | NA |
| pz_131 | F | 66 | Anoxic | 14 | VS/UWS | 7 | 5.88 |
| pz_132 | M | 38 | Traumatic | 252 | VS/UWS | 8 | 6.92 |
| pz_134 | F | 57 | Vascular | 5 | VS/UWS | 8 | 6.92 |
| pz_135 | M | 21 | Traumatic | 47 | MCS | 8 | 14.92 |
| pz_138 | M | 52 | Traumatic | 178 | MCS | 9 | 22.55 |
| pz_139 | F | 22 | Traumatic | 27 | MCS | 9 | 14.21 |
| pz_142 | F | 19 | Vascular | 6 | MCS | 12 | 39.22 |
| pz_143 | M | 61 | Traumatic | 103 | MCS | 10 | 23.59 |

**Supplementary Table 3**: Variables included in "Imaging" and "Imaging and Clinical" Variables for LowOrder and HighOrder networks. Abbreviations: sensorimotor (SM), auditory (AUD), lateral visual (LVIS), medial visual (MVIS), default mode network (DMN), salience (SAL), dorsal attention network (DAN), left and right fronto-parietal (L-FP, R-FP) and temporal (TEMP) network; AUC = area under the curve; Sens = sensitivity; Spec = specificity; C.I. = confidence interval; L = left; R = right. Acronyms are reported in Supplementary Table 1.

| ***LowOrder Networks*** | | | | | | | | |  |
| --- | --- | --- | --- | --- | --- | --- | --- | --- | --- |
|  |  |  |  |  |  |  |  |  |  |
| **Models with Imaging variables** | | | | | | | | |  |
|  | **Final model** | **AUC** | **Sens** | **Spec** | **C.I. AUC** | **Imaging variables (L/R)** | **VS/UWS misclassified  by etiology T/V/A** | **MCS misclassified  by etiology T/V/A** |  |
| **SM** | smc L | 0.64 | 0.88 | 0.24 | 0.48 0.64 | 1/0 | 5 3 4 | 9 9 4 |  |
| **AUD** | H-st L; H-st R | 0.62 | 0.83 | 0.12 | 0.46 0.60 | 1/1 | 5 4 5 | 10 12 5 |  |
| **LVIS** | iof L; iof R | 0.66 | 0.77 | 0.27 | 0.52 0.69 | 1/1 | 5 5 6 | 10 10 4 |  |
| **MVIS** | lc L; lc R | 0.66 | 0.80 | 0.29 | 0.49 0.66 | 1/1 | 5 6 4 | 8 10 4 |  |
| **All LowOrder** | smc L; iof L; lc R | 0.70 | 0.79 | 0.38 | 0.51 0.70 | 2/1 | 7 4 4 | 7 10 3 |  |
| **Models with Imaging and Clinical variables** | | | | | | | | |  |
| **SM** | Clinical variables; smc L | 0.73 | 0.79 | 0.44 | 0.66 0.84 | 1/0 | 5 6 5 | 6 7 4 |  |
| **AUD** | Clinical variables; H-st L; H-st R | 0.70 | 0.79 | 0.44 | 0.58 0.76 | 1/1 | 4 8 4 | 6 7 4 |  |
| **LVIS** | Clinical variables; iof L; iof R | 0.71 | 0.83 | 0.47 | 0.62 0.81 | 1/1 | 2 7 3 | 7 5 5 |  |
| **MVIS** | Clinical variables; lc L; lc R | 0.71 | 0.83 | 0.44 | 0.60 0.78 | 1/1 | 2 6 4 | 7 6 5 |  |
| **All LowOrder** | Clinical variables; smc L; iof L; lc R | 0.74 | 0.77 | 0.47 | 0.65 0.82 | 2/1 | 4 7 4 | 8 6 4 |  |
| ***HighOrder Networks*** | | | | | | | | |  |
|  |  |  |  |  |  |  |  |  |  |
| **Models with Imaging variables** | | | | | | | | |  |
|  | **Final model** | **AUC** | **Sens** | **Spec** | **C.I. AUC** | **Imaging variables (L/R)** | **VS/UWS misclassified  by etiology T/V/A** | **MCS misclassified  by etiology T/V/A** |  |
| **DMN** | lpc L | 0.63 | 0.89 | 0.32 | 0.53 0.70 | 1/0 | 1 4 2 | 7 13 3 |  |
| **SAL** | ai R; F2 R | 0.67 | 0.94 | 0.32 | 0.55 0.71 | 0/2 | 2 1 1 | 9 12 2 |  |
| **L-FP** | ilpc L | 0.64 | 0.82 | 0.38 | 0.51 0.70 | 1/0 | 5 5 2 | 5 13 3 |  |
| **R-FP** | ilpc R | 0.55 | 0.91 | 0.00 | 0.50 0.50 | 0/1 | 2 4 0 | 12 17 5 |  |
| **DAN** | ips L; ips R | 0.69 | 0.79 | 0.44 | 0.54 0.73 | 1/1 | 7 6 1 | 7 10 2 |  |
| **TEMP** | smt L; smt R | 0.62 | 0.85 | 0.18 | 0.46 0.62 | 1/1 | 4 2 4 | 10 14 4 |  |
| **All HighOrder** | dlpfc R; ai R; F2 L; ips L; lpc L;  mfc R; pcc R | 0.81 | 0.85 | 0.53 | 0.64 0.81 | 3/4 | 6 3 1 | 7 6 3 |  |
| **AllNetworks** | smc L; smc R; Heschl temp sup R;  lc R; dlpfc R; ai R; F2 L; ips L; mfc R; pcc R | 0.83 | 0.86 | 0.68 | 0.74 0.90 | 3/7 | 4 4 1 | 5 5 1 |  |
| **Models with Imaging and Clinical variables** | | | | | | | | |  |
| **DMN** | Clinical variables; lpc L; pcc L | 0.72 | 0.82 | 0.41 | 0.64 0.82 | 2/0 | 3 8 1 | 7 8 5 |  |
| **SAL** | Clinical variables; ai L; ai R; acc L; F2 L; F2 R | 0.75 | 0.83 | 0.53 | 0.65 0.83 | 2/2 | 5 6 0 | 6 6 4 |  |
| **L-FP** | Clinical variables; ilpc L | 0.73 | 0.8 | 0.41 | 0.61 0.79 | 1/0 | 4 8 1 | 6 9 5 |  |
| **R-FP** | Clinical variables; ilpc R | 0.69 | 0.85 | 0.35 | 0.55 0.73 | 0/1 | 1 8 1 | 9 8 5 |  |
| **DAN** | Clinical variables; ips L; ips R | 0.73 | 0.79 | 0.53 | 0.58 0.78 | 1/1 | 5 8 1 | 5 6 5 |  |
| **TEMP** | Clinical variables; smt L; smt R | 0.71 | 0.82 | 0.35 | 0.55 0.74 | 1/1 | 4 7 1 | 8 10 4 |  |
| **All HighOrder** | Clinical variables; dlpfc L; dlpfc R; smt L; ai R;  ips L; lpc L | 0.83 | 0.86 | 0.65 | 0.71 0.87 | 4/2 | 4 3 2 | 5 4 3 |  |
| **AllNetworks** | Clinical variables; smc L; lc R; dlpfc L; dlpfc R;  smt L; ai R; ips L; lpc L; mfc R; pcc R | 0.83 | 0.83 | 0.68 | 0.78 0.93 | 5/5 | 4 6 1 | 6 4 1 |  |
